# Connection between Inflammatory Connections of the Uteris and Its Displacements

**Published:** 1871-02

**Authors:** 


### Connection between Inflammatory connections of the Uteris and its Displacements.

Dr. J. Henry Bennett read an interesting paper on this subject before the Midwifery Section of the British Medical Association, August, 1870.

The following propositions express his views on the subject:

"1. I consider that, under the influence of mechanical doctrines pushed to an extreme, uterine displacements are by many too much studied *per se*, independently of the inflammatory and other lesions that complicate and often occasion them.

"2. That the examinations made to ascertain the existence of inflamatory complications are often not made with sufficient care and minuteness, as evidenced by the fact that I constantly see cases in practice in which inflammatory lesions have been neglected entirely, and in which the secondary displacements have been alone studied and treated.

"3. That inflammatory lesions are often the principal cause of uterine displacement through the enlargement and increased weight of the uterus, or of a portion of its tissues which it occasions.

"4. That when such inflammatory conditions do exist, as a rule, they should be treated and cured, and then time should be given to nature to absorb and reduce hypertrophied and engorged tissues before mechanical means of treatment are resorted to.

"5. That the relief from the sensation of bearing down which pessaries and bandages give is no real criterion of their being the proper means to use, such relief being often felt when there are inflammatory lesions present, which their presence aggravates.

"6. The above statements must not be considered in any way to imply that I do not recognise other causes of displacement of a non-inflammatory nature, such as laxity of ligaments and soft parts, wide pelvis, laceration of perineum, severe shocks, etc."—*British Medical Journal. American Journal, ibid.*

